# The End of a Classical Ontology for Quantum Mechanics?

**DOI:** 10.3390/e23010012

**Published:** 2020-12-24

**Authors:** Peter W. Evans

**Affiliations:** School of Historical and Philosophical Inquiry, University of Queensland, St Lucia, QLD 4072, Australia; p.evans@uq.edu.au

**Keywords:** causal symmetry, classical ontology, quantum foundations, contextuality, ontological models framework

## Abstract

In this paper, I argue that the Shrapnel–Costa no-go theorem undermines the last remaining viability of the view that the fundamental ontology of quantum mechanics is essentially classical: that is, the view that physical reality is underpinned by objectively real, counterfactually definite, uniquely spatiotemporally defined, local, dynamical entities with determinate valued properties, and where typically ‘quantum’ behaviour emerges as a function of our own in-principle ignorance of such entities. Call this view Einstein–Bell realism. One can show that the causally symmetric local hidden variable approach to interpreting quantum theory is the most natural interpretation that follows from Einstein–Bell realism, where causal symmetry plays a significant role in circumventing the nonclassical consequences of the traditional no-go theorems. However, Shrapnel and Costa argue that exotic causal structures, such as causal symmetry, are incapable of explaining quantum behaviour as arising as a result of noncontextual ontological properties of the world. This is particularly worrying for Einstein–Bell realism and classical ontology. In the first instance, the obvious consequence of the theorem is a straightforward rejection of Einstein–Bell realism. However, more than this, I argue that, even where there looks to be a possibility of accounting for contextual ontic variables within a causally symmetric framework, the cost of such an account undermines a key advantage of causal symmetry: that accepting causal symmetry is more economical than rejecting a classical ontology. Either way, it looks like we should give up on classical ontology.

## 1. Introduction

It should come as no surprise that many, perhaps even a good majority, of physicists after 1927 gave up on the view that the fundamental ontology of quantum mechanics is essentially classical: that is, the view that physical reality is underpinned by objectively real, counterfactually definite, uniquely spatiotemporally defined, local, dynamical entities with determinate valued properties, and where typically ‘quantum’ behaviour emerges as a function of our own in-principle ignorance of such entities. Let us call this position on the ontology of quantum theory Einstein–Bell realism. Despite the gloomy forecast for Einstein–Bell realists, it is well known that a class of responses to the canon of quantum no-go theorems, so-called causally symmetric local hidden variable approaches, plausibly rescues a large part of this classical picture of quantum theory. (I shall not be providing an argument for or against any particular causally symmetric local hidden variable approach here. See [1,2] for good reviews of such approaches. See also [3], in which we argue in favour of what we call the Price–Wharton approach). Indeed, causally symmetric local hidden variable approaches arguably comprise the last refuge for Einstein–Bell realism, positioned as they are to navigate a classical ontology through Bell’s theorem, the Kochen–Specker theorem, and the PBR theorem.

Part of the appeal of causal symmetry in this context is that it circumvents one of the integral assumptions of these three no-go theorems—that the properties of some quantum system have definite values independently of the measurement context to which the system is to be subject. With respect to Bell’s theorem, this admits local hidden variables—or local *beables*, as Bell called them—and with respect to the Kochen–Specker theorem, this not only admits noncontextual hidden variables, but also provides a natural explanation for why quantum systems appear to be contextual (as contextuality arises from the specific epistemic constraints of causal symmetry). A more recent no-go theorem, due to Shrapnel and Costa [4], undermines this case for noncontextual hidden variables. In short, the Shrapnel–Costa theorem removes the loophole open to ‘exotic causal structure’, and so implies that no ontological model, now including causally symmetric models, that satisfy the noncontextuality assumptions of the theorem can reproduce the statistical predictions of quantum mechanics. So in order to be a feasible model of such predictions, any causally symmetric ontology underpinning quantum behaviour must necessarily be contextual, along with the rest of the ontological models. Making matters worse is that the resulting form of the contextuality renders a natural explanation for this feature, as in the case of the Kochen–Specker theorem, much less plausible. In so far as causally symmetric local hidden variable approaches comprise the last refuge for Einstein–Bell realism, this contextuality is a concerning predicament for classical ontology. Indeed, I argue that this concerning predicament is as good as the end of a classical ontology for quantum mechanics.

The argument will proceed as follows. I begin in Section 2 by introducing the broad outline of causally symmetric local hidden variable approaches to the traditional no-go theorems, and I define Einstein–Bell realism. I focus in this section on contextuality, so introduce the ontological models framework and the operational formulation of the contextuality problem. In Section 3, I provide a brief outline of the history and development of the process matrix formalism and then go on to detail the Shrapnel–Costa theorem. I consider the three assumptions of the theorem that constrain ontological models that reproduce the statistical predictions of quantum mechanics—ω-mediation, instrument noncontextuality, and process noncontextuality—and briefly examine what a violation of each these assumptions implies. I consider in Section 4 what this means for Einstein–Bell realism and a classical ontology. I argue that, as a result of the Shrapnel–Costa theorem, the outlook is particularly worrying for Einstein–Bell realism and classical ontology. In the first instance, the obvious consequence of the theorem is a straightforward rejection of Einstein–Bell realism. However, more than this, I argue that, even where there looks to be a possibility of accounting for contextual ontic variables within a causally symmetric framework, the cost of such an account undermines a key advantage of causal symmetry: that accepting causal symmetry is more economical than rejecting a classical ontology. Either way, it looks like we should give up on classical ontology.

## 2. Causal Symmetry and Classical Ontology

### 2.1. The No-Go Theorems

Following the revolution of statistical mechanics in the late nineteenth century, one could surely be forgiven for expecting that the puzzles of quantum mechanics that emerged over the first few decades of the twentieth century would ultimately be explained by an underlying theory, comprised of hidden variables. This was a position for which Einstein, de Broglie, and others argued. It was not long before a no-go theorem jeopardising this position was proposed by von Neumann [5], who purported to show that the probabilistic nature of quantum theory could not be a function of an underlying theory of hidden variables. Bohm [6] showed by way of counterexample that there must have been something wrong with von Neumann’s theorem by proving that the predictions of his own hidden variable model were equivalent to the predictions of quantum theory. As a consequence of Bohm’s counterexample to von Neumann’s theorem, Bell developed a more precise no-go theorem—Bell’s theorem [7]—which clarifies that there can be no *local* hidden variable model that can match the predictions of quantum theory (and that satisfy the further reasonable assumptions of Bell’s theorem).

Bell’s comments on locality have attracted considerable attention (for instance, by [8,9], to name just two), but this detail should not concern us here. The basis of Bell’s assumption is that the specification of local beables at spacelike separation must be independent conditioned on their causal pasts. There is, however, another assumption in Bell’s theorem that is significant for our current purposes. This further assumption is known as *measurement independence*, and it requires that any hidden variables underlying a quantum system must remain statistically independent of the choice of measurement settings to which that system is subject as part of the experimental procedure [1]. If one were to reject the assumption of measurement independence, one could maintain Bell’s assumption of locality by permitting beables to be correlated explicitly with the choice of measurement setting, even if that choice occurs in the future of the quantum system. (Another, metaphysically distinct, way to reject the assumption of measurement independence is to remove the freedom of agents to choose measurement settings arbitrarily, leading to superdeterministic local hidden variables approaches [8,10,11]. Causal symmetry is generally more favourable than superdeterminism on account of the fact that one can explicitly preserve the free choice of agents by rejecting the assumption of strictly forwards-in-time causality.) Such a correlation can then be accounted for by giving up the conventional assumption that causation must occur only in the forward temporal direction, such that the choice of measurement setting might causally influence the hidden variables underlying the quantum state in the backward temporal direction. Thus, the resulting causal symmetry denies one of the assumptions underpinning Bell’s theorem, measurement independence, without violating the assumption of locality. This move rescues the possibility of local hidden variables, so long as one admits symmetric causal influences, both forwards and backwards in time.

Very shortly after Bell’s theorem followed the Kochen–Specker theorem [12], which will be particularly relevant for our discussion below. This theorem states that any hidden variable model that reproduces the predictions of quantum theory must be *contextual*. A model or theory is said to be contextual when the properties attributed to some system described by the theory are dependent upon the means of realising some value for that property, or *context* of measurement or observation of those properties, beyond the actual observation itself. Thus, we can think of a *noncontextual* ontological property as one that can be unambiguously distinguished experimentally. A relevant example of a context of measurement beyond the measurement itself would be the set of further properties, if any, that are measured in conjunction with the measurement. Since in classical mechanics there is no such dependence on measurement context for properties attributed to systems as a result of some measurement, classical mechanics is noncontextual. According to the Kochen–Specker theorem, any model of a quantum system that assumes that measurements deterministically uncover the values of pre-existing dynamical variables—such as beables—must be contextual. So, just as Bell’s theorem shows that there can be no *local* hidden variable model satisfying his assumptions that can match the predictions of quantum theory, the Kochen–Specker theorem shows that there can be no noncontextual deterministic local hidden variable model that can match the predictions of quantum theory.

However, just as we saw above that rejecting Bell’s assumption of measurement independence, and so permitting correlations that arise as a result of symmetric causal influences, rescues local hidden variables from Bell’s theorem, hypothesising such causal symmetry allows a similar rejection of the assumption of measurement independence for the Kochen–Specker theorem. Under the assumption that the correlation between the value of any pre-measurement hidden variables and the context of measurement can be explained by a causal influence directed from the latter to the former, we can take the measurement process to be *bringing about* the determinate valued properties that constitute the hidden variables, rather than uncovering independently existing variables [1]. This account has the added bonus of providing a ‘natural’ explanation for why quantum theory is contextual: the dependence of any measured values on the eventual context of measurement should be expected in a regime that admits symmetric causal influences. This is because the natural epistemic constraints that arise when describing a physical system whose complete description depends upon a future boundary condition result in an impoverished, statistical description of the system conditioned only upon the initial boundary condition (see, for instance, [13]). Indeed, Wharton [14] (p. 203) suggests that contextuality just is the failure of measurement independence.

So far this brief historical narrative is straightforward. However, since contextuality is at the heart of the Shrapnel–Costa theorem, we need to develop further layers to this narrative. The particular way of understanding contextuality adopted by Shrapnel and Costa has its origin in the work of Spekkens [15]. To get to grips with this treatment of contextuality, we need to say a few words about the ontological models framework.

### 2.2. Ontological Models

The ontological models framework was first introduced in [15], and developed further in [16] (see also [17,18] for good reviews), and provides a formalisation of our notion of classical ontology in an operational model of quantum processes [1]. As an operational model, the framework specifies a set of possible preparations, transformations, and measurements, and associated outcome probabilities, to describe the observed statistics over the possible outcomes. In addition, the framework also specifies an ontological model to account for the observed statistics in the following way.

The quantum state, ψ, is the description we give to the quantum system after the preparation procedure. We assume, however, that we can completely specify the properties of the quantum system as a result of preparation in terms of its actual *ontic* state, λ, which arises via a classical probability density, $\mu_{\psi }\left(\lambda \right)$, over the set of possible ontic states, Λ. We can then specify the outcome probabilities for each measurement procedure, *M*, conditional on λ, such that the outcomes, {m}, are independent of the preparation procedure. We say that λ*screens-off* the preparation from the measurement so that measurement outcomes only depend on the ontic state. Leifer and Pusey [19] call this feature *λ-mediation*, as the ontic state completely mediates any correlations between preparation procedures and measurement outcomes. Importantly for our narrative here is that, explicitly according to the ontological models framework, λ does not causally depend on the future choice of measurement procedure, *M*,

(1) P(λ∣M)=P(λ),

otherwise λ could not screen off the {m} from ψ. Finally, as a result of this setup, the eventual operational statistics given by the conditional outcome probabilities need to reproduce the quantum statistics.

Let us add to this a useful distinction from the ontological models framework between what Harrigan and Spekkens [16] call ψ-ontic and ψ-epistemic interpretations of the wavefunction. In a ψ-ontic interpretation, each ontic state is consistent with a single quantum state. In a ψ-epistemic interpretation, multiple distinct quantum states can be consistent with a single ontic state. A further orthogonal distinction can be overlaid across this. A ψ-complete interpretation takes the quantum state to provide a complete description of objective reality (that is, there is a one-to-one correspondence between quantum states and ontic states), while a ψ-incomplete interpretation requires that the quantum state be supplemented with additional ontic degrees of freedom (such as, for instance, in typical ψ-ontic pilot-wave interpretations). Given this latter distinction, ψ-epistemic interpretations are naturally ψ-incomplete, and render the wavefunction a representation of the knowledge of the user of the formalism rather than a representation of objective reality. For ψ-epistemic interpretations in the ontological models framework, there exists an ontic state underlying the wavefunction. (Beyond the ontological models framework, a ψ-epistemic wavefunction can be given an anti-realist or operationalist interpretation that makes no such claim for a deeper underlying objective reality.)

We can apply this framework to our analysis of Bell’s theorem above. The ontological models framework is explicit that λ does not causally depend on the measurement procedure (1), which is, of course, simply Bell’s assumption of measurement independence. As we saw above, by denying this assumption and accounting for the correlation between hidden variables, or the ontic state λ, and the measurement procedure by way of causal symmetry, we can rescue the assumption that any beables are local. As a result of this move, then, we can see that the admission of causal symmetry is a ready made violation of the assumption of a strict temporal and causal order for the ontological models framework [18] (p. 94).

As a very brief aside, and before we continue with our explication of contextuality in quantum theory, we now have the tools to summarily deal with the causal symmetry response to the PBR theorem [20]. The PBR theorem states, in short, that the ontic states of any interpretation of quantum mechanics that fits within the Bell framework and reproduces the Born rule must be in one-to-one correspondence with the quantum states. This, of course, is simply the definition of a ψ-ontic interpretation of the wavefunction. Thus, the PBR theorem appears to be ruling out ψ-epistemic approaches to the wavefunction. However, since the PBR theorem is framed in the ontological models framework, it is straightforward to note as before that it does not apply to causally symmetric approaches. Just as with Bell’s theorem, admitting causal symmetry rescues the possibility of an interpretation of the wavefunction as an epistemic representation of an underlying reality of local beables.

Let us return then to contextuality. Spekkens [15] pioneered a new way of thinking of contextuality beyond the formulation of [12]. According to Spekkens’ characterisation, an ontological model of an operational theory is *noncontextual* when operationally equivalent experimental procedures have equivalent representations in the ontological model [15] (p. 1). Thus, contextuality on this account implies that operationally equivalent experimental preparation procedures may correspond to inequivalent ontic state representations (and where the state additionally depends on the context of measurement). Defining contextuality in this way, rather than in terms of the deterministic uncovering of the values of pre-existing dynamical variables, renders the assumption of noncontextuality as a principle of parsimony: no ontological difference without operational difference. (Spekkens [21] argues that this generalised noncontextuality is an embodiment of Leibniz’ Principle of the Identity of Indiscernibles, and labels this principle the *Leibnizian methodological principle*. Schmid et al. [22] formalise this principle under the term *Leibnizianity*. We consider this principle again in Section 4.) Despite this difference in flavour, Spekkens’ diagnosis of hidden variable approaches concludes with the same result. Assuming that there are ontic properties underlying the observed statistical behaviour of quantum systems, and that the associated ontic states are distinct just when there are corresponding operational differences, cannot account for the statistical behaviour entailed by quantum theory. The result is then that there is no noncontextual ontological model that can reproduce the observed statistics of quantum theory.

Just as we saw above with respect to Bell’s theorem and the PBR theorem, however, in so far as the no-go theorems can be characterised in the ontological models framework, the no-go theorems are undermined by the assumption of causal symmetry. Thus Spekkens’ more general understanding of contextuality in terms of the ontological models framework suffers the same response: causal symmetry is a categorical rejection of the assumption of a strict temporal and causal order that underpins the ontological models framework. Noting this is the main result of this section of the paper: in so far as there is a motivation to avoid the consequences of the above no-go theorems, particularly to maintain some semblance of a classical ontology, hypothesising causal symmetry is a strategy that is well placed to achieve this.

The no-go theorems are ordinarily taken to demonstrate that the underlying conceptual and ontological framework of quantum theory cannot be completely classical, and cannot be about local, noncontextual hidden variables. Any hope one might have for hanging on to such a classical ontology rests with causally symmetric local hidden variable approaches, as per our story here. Whilst such approaches are admittedly unorthodox, and even perhaps in a sense ‘nonclassical’ on account of causal symmetry, what is significant about such approaches is that they have the potential to rescue an objectively real, counterfactually definite, uniquely spatiotemporally defined, local, noncontextual (or where any contextuality is underpinned by noncontextual epistemic constraints), determinate valued ontology, where typically ‘quantum’ behaviour emerges as a function of our own in-principle ignorance of such entities [1].

As I propose in [23] (and also in [1]), the distinction that Quine [24] draws between the ‘ontology’ and the ‘ideology’ of a theory is an apt distinction to understand the value of the above argument. According to Quine, the ideology of a theory is comprised of the set of ideas that can be expressed by a theory, and thus ideological economy is then a measure of the economy of primitive undefined statements employed to reproduce this ideology. Put in these terms, one of the consequences of the above narrative is that the ideology of causal symmetry is more economical than a rejection of classical ontology. The main goal of the current work is to make clear how the Shrapnel–Costa no-go theorem significantly jeopardises this position. In short, motivated by the ‘get out of jail free’ card that causal symmetry is able to play with respect to the no-go theorems, Shrapnel and Costa develop a no-go theorem that closes off the ‘measurement independence’ loophole to any approach that assumes an ‘exotic’ form of causality.

Before we consider the Shrapnel–Costa theorem in Section 3, let us once more explicitly state our characterisation of classical ontology by adopting the framework which my collaborators and I have elsewhere called Einstein–Bell realism [3], bringing together the above analysis of the no-go theorems.

### 2.3. Einstein–Bell Realism

Consider the following conditions from [3], called the *Einstein–Bell conditions*, adapted here to nonrelativistic quantum theory:

1. Quantum mechanical probabilities are epistemic;
2. Quantum mechanics is local;
3. Quantum mechanics is consistent with the no-go theorems.

We can think of Einstein–Bell realism as the conjunction of these conditions with the assumption that there is an objective reality.

There is good reason for calling this view Einstein–Bell realism. It should be clear to see that the first condition epitomises the sentiment that Einstein expressed to Born in 1926 that “God does not play dice” (to be clear, I take this as a statement of determinacy rather than determinism). This also has implications for any ensuing interpretation of the wavefunction. Consider the distinction above between ψ-ontic and ψ-epistemic interpretations of the wavefunction. Harrigan and Spekkens [16] argue that Einstein, in his more sophisticated arguments for the incompleteness of quantum mechanics, advocated for a realist ψ-epistemic interpretation. Despite the fact that there are some ψ-ontic, ψ-incomplete interpretations—like pilot-wave interpretations—in which probabilities arise as a function of our ignorance over the full ontic degrees of freedom, and so are epistemic, the basic probabilistic concepts that occur in all ψ-epistemic interpretations are clearly also themselves epistemic. Thus, ψ-epistemic interpretations are natural bedfellows for epistemic notions of probability [3] (p. 6).

The second assumption epitomises not only Einstein’s implicit appeal to consistency between quantum theory and relativity (as in, for instance, [25]), but also the exclusion of spacelike separated influences formalised in Bell’s own theorem (as in, for instance, [26]). For clarity, and following [3] (p. 5), we can take the assumption here to mean that physical bodies and influences can only follow timelike or null spacetime trajectories, and so these trajectories must obey Lorentz covariance. The third assumption reflects that a plausible interpretation of quantum theory must obey the constraints established by the above no-go results, that is, from Bell’s theorem, the Kochen–Specker theorem, and the PBR theorem.

Employing the Einstein–Bell conditions, we argue in [3] that causally symmetric local hidden variable approaches constitute a unique realist interpretation satisfying these constraints. Our logic there is straightforward. The first condition requires a ψ-incomplete interpretation, as epistemic probabilities cannot be accommodated by ψ-complete interpretations. The second and third conditions together exclude ψ-ontic interpretations, as such interpretations cannot be local according to the no-go theorems, implying that only ψ-epistemic interpretations can meet the Einstein–Bell conditions. Furthermore, a causally symmetric approach circumvents the results of Bell’s theorem, and so can be a local hidden variable theory, also circumvents the PBR theorem, and so permits a ψ-epistemic wavefunction, and also contains an explicit contextuality of the ontic state on the experimental procedure, so fits within the bounds given by the Kochen–Specker theorem. So we can see that the commitments of Einstein–Bell realism capture what we mean by local beables, or by noncontextual local hidden variables: a commitment to a local-realist ontology underlying a ψ-epistemic wavefunction. Causally symmetric hidden variable approaches are, according to the arguments in [3], the only approaches that fit these constraints. As we will now see, however, these constraints have just gotten prohibitively tighter.

## 3. The Shrapnel–Costa No-Go Theorem

Shrapnel and Costa [4] argue that exotic causal structures—such as causal symmetry—are incapable of explaining quantum behaviour arising as a result of noncontextual ontological properties of the world. As we have just seen, one of the key underlying assumptions of the Kochen–Specker theorem is that quantum phenomena arise on a fixed background forwards-in-time causal structure, and so do not preclude the possibility of more exotic causal structures providing an ontologically classical (noncontextual) explanation. This leaves open the possibility of symmetric causal structure allowing noncontextual ontological properties to underpin quantum behaviour, and also providing a natural explanation for *why* typically quantum behaviour can arise from such a noncontextual ontology.

The Shrapnel–Costa theorem is stronger than the Kochen–Specker theorem, as it closes off the possibility of exotic causal structure providing just such a noncontextual explanation of quantum behaviour. In fact, it shows that *any* ontology underpinning quantum behaviour must be contextual; moreover, “what is contextual is not just the traditional notion of “state”, but any supposedly objective feature of the theory, such as a dynamical law or boundary condition, which is responsible for the experimentally observed statistics” [4] (p. 2). In order to take account of the possibility of exotic causal structure, the Shrapnel–Costa theorem first generalises the ontological models framework, and then employs the *process matrix formalism*, which is suited to describing processes with indefinite causal structure. To get to the heart of the Shrapnel–Costa theorem, then, let us begin by reviewing the process matrix formalism.

### 3.1. Process Matrix Formalism

The development of the process matrix formalism is punctuated by a number of independent redevelopments. The roots of the formalism stretch back to [27,28] on the dynamics of open quantum systems and quantum stochastic processes in the 1970s. This early work is framed in the language of algebraic quantum field theory. The development of quantum information theory at the beginning of this century allowed for some of the ideas corresponding to those from the dynamics of open quantum systems to be recast into a modern form, whereby, for instance, ‘non-Markovian quantum stochastic processes’ has become ‘quantum channels with memory’. In the field of experimental quantum information, process matrices began to be popularised in the analysis of quantum process tomography of quantum optical systems (see, for instance, [29,30,31,32,33])—whereby processes matrices were usually obtained experimentally from quantum state tomography [29]. However, much of the contemporary discussion regarding the process matrix formalism can be traced back to the seminal work [34], couched in the language of quantum information theory, and developed largely independently of the earlier work on open quantum systems.

In short, a process matrix—to be defined below—is a way of representing the state transformation denoted by the completely positive map, $M^{A}\left(\rho \right)$, generated by the evolution of a quantum state, ρ, from an input state space, $H^{A_{1}}$, to an output state space, $H^{A_{2}}$, by some quantum operation, $M^{A}:L\left(H^{A_{1}}\right)\to L\left(H^{A_{2}}\right)$. A key step to developing the process matrix formalism is the realisation that the Choi–Jamiołkowski isomorphism [35,36], which establishes a correspondence between linear maps and linear operators, allows the linear map $M^{A}:L\left(H^{A_{1}}\right)\to L\left(H^{A_{2}}\right)$ to be conveniently rewritten as a linear operator $M^{A_{1}A_{2}}\in L\left(H^{A_{1}}⊗H^{A_{2}}\right)$ (see [37] in this issue for more on the interpretation of the Choi–Jamiołkowski isomorphism). Following on from [34], the ‘Pavia group’ employed this Choi–Jamiołkowski representation to describe transformations on a network of quantum gates, and developed a methodology for optimising such *quantum circuits* using what they call a *quantum comb*: a temporally ordered physical process with a quantum memory [38,39]. (Incidentally, the quantum comb has been independently redeveloped no less than two more times [40,41].) They further demonstrated that simple quantum circuits can be physical implementations of what they call *quantum supermaps*, mapping an input quantum operation to an output quantum operation [42].

At about the same time, and independently, Gutoski and Watrous [43] also employed the Choi–Jamiołkowski representation to represent what they call *quantum strategies*: a specification of the exchange and processing of quantum information in a quantum process. This development is largely equivalent to the quantum comb (along with the operator tensor formulation of quantum theory [44], which contains close similarities). The Pavia group, however, went on in the ensuing years to develop and analyse the quantum control of temporal order in the *quantum switch*, where two operations are enacted in a quantum superposition of the two possible temporal orders [45,46,47]. The significance of the quantum switch here is that the process matrix formalism developed as the ideal formal system for describing such indefinite causal order.

Hardy [48] introduces the notion of indefinite causal structure in the context of quantum gravity, and Oeckl [49], also motivated by quantum gravity, considers a “general boundary” formulation of quantum mechanics that does not assume an a priori causal structure (and which overlaps significantly in the quantum context with the two-state vector formalism [50]). However, it is Oreshkov et al. [51] who analyse indefinite causal order in the context of quantum processes: they employ the Choi–Jamiołkowski representation, and a local causal direction, to derive a causal inequality violated by timelike and spacelike correlations with a global causal direction. It is this work on process matrices and indefinite causal order that has in part led to the development of quantum causal modelling [52,53,54,55], the framework within which Shrapnel and Costa build their no-go theorem.

The utility of the process matrix formalism is that it provides a framework to describe communication tasks between parties that lack a definite causal order. Given a set of parties each residing in their own laboratory, A,B,…, one assumes that each party is able to act locally on some system that passes once through their laboratory, where local operations are described by ordinary quantum mechanics. No assumption is made concerning the relative spatiotemporal arrangement of the laboratories, nor that there is ultimately some causal structure within which the laboratories are positioned.

Following [51], the operations that any such party can perform are delineated by a *quantum instrument*, $M^{A}$, which induces—by way of a unitary transformation and projective measurement—a transformation from input to output, $M_{i}^{A}$, indexed by the *outcome*, i=1,…,n. $M_{i}^{A}$ is a completely positive (CP) trace-non-increasing map. The set of all CP maps for some instrument—which when taken together, ${\left\{M_{i}^{A}\right\}}_{i=1}^{n}\equiv M^{A}$, exemplify the fact that with probability one there must be some outcome of the application of the instrument—is a CP and trace-preserving (CPTP) map.

For a set of parties, the set of all outcomes across the laboratories corresponds to a set of CP maps, $M_{i}^{A},M_{j}^{B},\ldots$, and the complete list of probabilities $P(M_{i}^{A},M_{j}^{B},\ldots )$ for all i,j,… is what Oreshkov et al. [51] (p. 3) call a *process*. The process thus exemplifies all the operational correlations between local laboratories. An important assumption that Oreshkov et al. make at this point is that each such joint probability is noncontextual, in the sense that the joint probability for any set of CP maps is not dependent on the local detail of any particular instrument $M^{A}$. It is most obvious here in this characterisation of ‘process’ the way in which it can be seen as a generalisation of ‘state’: both can be seen as encoding a list of probabilities over a set of measurement scenarios.

Since, as above, the linear maps $M_{i}^{A}$ and $M_{j}^{B}$ can be represented, using the Choi–Jamiołkowski isomorphism, by the linear operators $M_{i}^{A_{1}A_{2}}$ and $M_{j}^{B_{1}B_{2}}$, respectively, the joint probability for two measurement outcomes (*i* at *A* and *j* at *B*) can be expressed as a function of the corresponding Choi–Jamiołkowski operators [51] (p. 4)]:

(2) $$P\left(M_{i}^{A},M_{j}^{B}\right)=\mathrm{Tr}[W^{A_{1}A_{2}B_{1}B_{2}}\left(M_{i}^{A_{1}A_{2}}⊗M_{j}^{B_{1}B_{2}}\right)].$$

Here, $W^{A_{1}A_{2}B_{1}B_{2}}$ is known as the *process matrix*, under the condition that it is positive semi-definite ($W^{A_{1}A_{2}B_{1}B_{2}}\geq 0$)—which embodies the constraint that the instruments $M^{A}$ and $M^{B}$ are CPTP maps—and its trace is one [56].

The process matrix is readily understood as a generalisation of a density matrix—and so can be seen as a generalisation and extension of the notion of state—and the trace rule (2) as a generalisation of the Born rule [51]. Further, more restrictive assumptions allow one to reduce the process matrix to the quantum state (when the output systems are one-dimensional), to reflect the characterisation of a quantum comb or network (when a definite causal order is fixed), or to represent quantum channels with memory (when only unidirectional signalling is possible).

With this brief survey of the process matrix formalism, let us turn our attention to the Shrapnel–Costa no-go theorem.

### 3.2. Causation Does Not Explain Contextuality

Shrapnel and Costa begin by outlining a generalised operational framework to reflect the ontological models framework of [15]. However, they are interested in replacing the operational notions of preparation, transformation, and measurement procedures with more temporally and causally neutral concepts. They replace these operations with the general notion of *local controllables*, ${\tilde{I}}^{A}$, where each local controllable is indexed to a *local region*, A,B,…, and each choice of local controllable is labelled by an *outcome*, a,b,…. Furthermore, the physical features of the world external to the system, and independent of the choice of local controllables, including “any global properties, initial states, connecting mechanisms, causal influence, or global dynamics”, responsible for correlating outcomes between local regions they call the *environment*, $\tilde{W}$. Importantly, any variable correlated with the choice of local controllable is necessarily considered an outcome and cannot be part of the environment [4] (p. 5).

They define an *event*, $M^{A}$: =$\left[\left(a,{\tilde{I}}^{A}\right)\right]$, as an operational equivalence class of pairs of outcome and local controllable, such that the joint probabilities over outcomes are equivalent for all possible outcomes and local controllables in all the other regions and for all environments [4] (p. 7). In the process matrix framework (that is, in the quantum context) this is analogous to the role played by the CP maps $M_{i}^{A}$, which we can call here *quantum events*. They define an *instrument*, $I^{A}$, similarly as an operational equivalence class of lists of possible (that is, with non-zero probability) events, $M^{A}$; that is, $I^{A}$: =$\left\{M_{i}^{A}\right\}$. This is analogous to the role played by the CPTP maps $M^{A}$ in the process matrix formulation, which recall are labelled *quantum instruments*.

Moreover, just as we noted above that the joint probability $P(M_{i}^{A},M_{j}^{B},\ldots )$ is noncontextual, in the sense that it is not dependent on the local detail of any particular instrument $M^{A}$, the probability of some event $M^{A}$, so long as $I^{A}$ renders $M^{A}$ possible, is independent of the particular instrument I. Thus, as above, correlations between events across different regions are not a function the details of the instrument, which itself specifies events that do not happen, and so events screen-off instruments. Shrapnel and Costa call this *operational instrument equivalence* and note that it is equivalent to noncontextuality in the former sense [4] (p. 13).

Finally, they define a process, *W*: =$\left[\tilde{W}\right]$, as an operational equivalence class of environments, such that the joint probabilities over outcomes given local controllables are equivalent across the equivalence class of environments. In the quantum context, *W* is the *process matrix*. This generalisation of outcomes, local controllables, and the environment into events, instruments, and the process serves to operationalise any joint probability distribution to allow the creation of an ontological model—along the lines of the ontological models framework—that underlies the distributions that we take to account for the observed statistics.

The major shift enacted by Shrapnel and Costa from the ontological models framework of [15,16] to ontological models in the process matrix framework is to move away from the idea that the ‘state’ encodes the ontology of some system towards the idea that more general properties of the environment are responsible for mediating correlations between the regions. As such, they replace the ontic state λ with the *ontic process* ω:

our ontic process captures the physical properties of the world that remain invariant under our local operations. That is, although we allow local properties to *change* under specific operations, we wish our *ontic process* to capture those aspects of reality that are independent of this probing.

[4] (p. 8)

Those aspects of reality that the ontic process captures are those parts of the environment that are not within the control of the experimenters, like initial conditions, causal influences, and global dynamics.

Shrapnel and Costa then make three natural assumptions that they take an ontological model in their framework to obey. Firstly, they replace the notion of λ-mediation (as per [19] above) with the notion of *ω-mediation*, in which the ontic process ω completely specifies the properties of the environment that mediate correlations between regions, and screens off outcomes produced by local controllables from the rest of the environment [4] (p. 8):

(3) $$P\left(a,b,\ldots |{\tilde{I}}^{A},{\tilde{I}}^{B},\ldots \tilde{W}\right)=\int d\omega P\left(a,b,\ldots |{\tilde{I}}^{A},{\tilde{I}}^{B},\ldots \right)P\left(\omega |\tilde{W}\right).$$

Secondly, they define the notion of *instrument noncontextuality* as a law of parsimony (much like [15]): operationally indistinguishable pairs $\left(a,{\tilde{I}}^{A}\right)$, $\left(a^{\prime },{\tilde{I^{\prime }}}^{A}\right)$ should remain ontologically indistinguishable. That is, $\forall b,c,\ldots {\tilde{I}}^{B},{\tilde{I}}^{C},\ldots ,\omega$ [4] (p. 9):

(4) $$P\left(a,b,\ldots |\omega ,{\tilde{I}}^{A},{\tilde{I}}^{B},\ldots \right)=P\left(a^{\prime },b,\ldots |\omega ,{\tilde{I^{\prime }}}^{A},{\tilde{I}}^{B},\ldots \right).$$

This allows them to define a probability distribution on the space of events, conditional on instruments and the ontic process, $P(M^{A},M^{B},\ldots |\omega ,I^{A},I^{B},\ldots )$, in terms of a function that maps events to probabilities. As Shrapnel and Costa point out, instrument noncontextuality is formally identical to operational instrument equivalence, except for the fact that instrument noncontextuality includes the ontic process.

Thirdly, they define the notion of *process noncontextuality*: operationally indistinguishable $\tilde{W}$, ${\tilde{W}}^{\prime }$ should remain ontologically indistinguishable [4] (p. 9).

(5) $$P\left(\omega |\tilde{W}\right)=P\left(\omega |{\tilde{W}}^{\prime }\right).$$

Again, this allows them to define a probability distribution on the space of ontic processes, in terms of a function that maps ontic processes to probabilities.

The Shrapnel–Costa no-go theorem is then that there can be no ontological model that satisfies ω-mediation, instrument noncontextuality, and process noncontextuality. They argue as follows. As we have just noted, each of instrument and process noncontextuality defines a function that maps from the space of events, $\left\{M^{A},M^{B},\ldots \right\}$, and ontic processes, ω, respectively, to probabilities. However, the two noncontextuality assumptions force these functions to be ordinary positive probability distributions. Since quantum expectation values cannot be expressed in this way, no instrument and process noncontextual ontological model can reproduce the quantum statistical predictions.

### 3.3. Interpreting the Result

So what does this result mean, exactly? Well, to begin with, beyond pointing out the intended consequence of their theorem, Shrapnel and Costa do not speculate on further consequences in any great detail. The intended consequence is that, since preparations, transformations, and measurements have been replaced by local controllables, there is no further assumption in the no-go theorem that ω is correlated with some controllables but independent of others. Recall that this is the form of the ‘loophole’ in the orthodox ontological models framework through which we are able to thread causally symmetric local hidden variable approaches to defeat the nonclassical consequences of the no-go theorems from Section 2. The part of the theorem doing most of the heavy lifting on this point is ω-mediation. By replacing λ-mediation with ω-mediation, the relevant correlations are not simply a function of the quantum state, but the agent-independent rules or laws that we take the environment to contribute to the dynamical behaviour of a system, and the connection between local action and observed events. Where causal symmetry is ideally placed to circumvent λ-mediation, no such causal assumption can do so for ω-mediation. Thus, this loophole is closed off in the Shrapnel–Costa theorem, rendering causally symmetric approaches just as contextual as the rest of the models captured by the ontological models framework [1]. So causally symmetric local hidden variable approaches, on account of being ontological models, must violate one of the assumptions of the Shrapnel–Costa theorem to hope to match the statistical predictions of quantum mechanics (and superdeterministic hidden variable models, also on account of being ontological models, fare no better at meeting this challenge). In so far as this sets a challenge to causally symmetric approaches, the discussion in the next section explores the possibility of meeting this challenge. To this end, while Shrapnel and Costa note only briefly the consequences of violating each of their assumptions, let us consider precisely what such violations entail.

The consequences of violations of the assumption of ω-mediation are not limited to transgressions against a realist attitude towards the ontology of quantum systems. As we noted just above, the assumption of ω-mediation insinuates that there are observer-independent aspects of the world, such as boundary conditions and global dynamics, that are ‘there’ to be discovered by the experimental procedure. Violations of this assumption do not just offend realist attitudes towards the state, then, but would require a radical rethink of the nature of scientific inquiry and our role as observers in that process.

Shrapnel and Costa have notable things to say about the assumption of instrument contextuality. Firstly, it is interesting to note the possibilities that they consider in which ontological models that satisfy instrument noncontextuality could be interpreted, from an ordinarily time-oriented perspective, as contextual [4] (pp. 11–13). This is the same phenomenon at play as the one employed by causally symmetric approaches with respect to measurement noncontextuality. As we saw above in Section 2.1, the ‘added bonus’ of causally symmetric approaches to contextuality in the Kochen–Specker theorem was that it provided a natural explanation for this contextuality in terms of epistemic constraints that arise from the ordinary temporal orientation of observers. However, the nonextendibility result in the case of measurement noncontextuality—that no noncontextual extension of quantum theory can provide more accurate predictions of outcomes [57]—holds in the case of instrument noncontextuality, too. That is, no instrument noncontextual hidden variable can provide more information than is contained in the process matrix [4] (p. 17). This result rules out nontrivial hidden variable extensions such that obtaining greater predictive power for quantum theory can only be achieved by the addition of *contextual* variables.

Violations of the assumption of instrument noncontextuality would imply that correlations between events across different regions depend upon the details of the quantum instrument—in particular, on the CP maps that are not employed as part of the choice of local controllable—and so on events that do not in fact happen. Interestingly, this would imply that events do not screen-off instruments, and so lends weight to the idea that contextuality is a species of fine-tuning [58]. This flavour of noncontextuality has strong similarities to preparation and measurement noncontextuality, and thus the ontological consequences of violations of these have received considerable attention already (see, for instance, [17,59]). Since these do not represent a particularly novel type of consequence for the Shrapnel–Costa theorem, I will not labour these consequences here.

The consequences of violations of the assumption of process noncontextuality are certainly novel. Process contextuality implies that operationally equivalent arrangements of an experiment do not necessarily lead to equivalent ontic descriptions of that experiment. What is significant about this is that included in this ontic description are

all aspects of a physical scenario other than the choices of settings and the observed outcomes…Such aspects include what kind of systems are involved, the laws describing such systems, boundary conditions, etc.

[4] (p. 18)

Thus, it seems that process contextuality would have a devastating effect on our ability to conduct orthodox scientific inquiry. The implication here is that operationally equivalent experimental arrangements may be realised by inequivalent ontic states, global properties, causal mechanisms, laws, or boundary conditions—indeed, any part of the environment that is not within the control of the experimenters. It is difficult to imagine exactly what this means, ontologically speaking. However, I speculate in the next section that one solution to a previously identified problem with a particular causally symmetric approach may provide a suggestion as to what process contextuality could amount.

## 4. The End of a Classical Ontology?

We are now in a position to assess the prospects for causally symmetric local hidden variable approaches to quantum theory in the face of the Shrapnel–Costa theorem and, in so far as such approaches are the only approaches that fit the constraints of Einstein–Bell realism, with them the prospects for Einstein–Bell realism and a classical ontology. To some, the loss of the possibility of a classical ontology is surely no news at all; as I noted in the opening sentence to this work, many physicists gave up on the view that the fundamental ontology of quantum mechanics is essentially classical after 1927, and certainly after the Bohr-Einstein debates ran their course beyond 1935 [25,60].

Thus the first obvious option in response to the Shrapnel–Costa theorem is to accept that any rescue effort for Einstein–Bell realism is now determinately impossible. If one wanted, one could still maintain that the wavefunction be interpreted as ψ-epistemic, so long as there were no classical ontology underlying the wavefunction description (as in some versions of QBism [61]). Alternatively, one could maintain a realist attitude towards the quantum formalism by adopting a ψ-ontic interpretation, with or without additional ontic degrees of freedom. Perhaps the clearest avenue here is a ψ-ontic wavefunction without additional ontic structure (such as the many-worlds interpretation [62]), as one must keep in mind that any additional ontic degrees of freedom would need to be nonlocal and/or contextual.

However, is there a way to rescue Einstein–Bell realism? I see only two remaining options. The first is to undermine the Shrapnel–Costa theorem, possibly by rejecting one or more underlying assumptions. It is a difficult task to identify the most appropriate foundations to challenge, but perhaps rethinking the nature of causality, inference, and/or probability might be a fruitful place to begin searching. (Hofer-Szabó [63] considers the possibility of giving up on Spekkens’ definition of generalised noncontextuality, which also underpins the Shrapnel–Costa theorem, and simply permitting unproblematically the possibility of a quantum system responding differently to different measurements represented by the same operator.)

Schmid et al. [22] propose a generalisation of causality and inference—what they call a *causal-inferential theory*—with the express intention of avoiding the consequences of the traditional no-go theorems that rule out local or noncontextual realist approaches. They make the suggestion that previous such generalisations that can be employed to provide diagnoses of the true consequences of the no-go theorems “scramble” the ontological and epistemological aspects of this problem. The previous generalisations they mention include the ontological models framework from above [15], quantum generalisations of propositional logic (‘quantum logic’ [64,65,66]), operational probability theories (which originate from the Pavia group [67]), generalised probability theories (beyond quantum logic [68]), and a nonclassical generalisation of Bayesian inference [52]. What is significant about this proposal is the way that Schmid et al. are explicitly attempting to “salvage” the notions of locality and noncontextuality, and so salvage, at least in part, a classical ontology. A rough outline of their main conjecture is as follows.

Recall that in Section 2.2 we were introduced to the term *Leibnizianity* that Schmid et al. employ to characterise generalised noncontextuality. Their formalisation simply captures the proclamation that there should be no ontological difference without operational difference. They claim that the traditional noncontextuality no-go theorems constrain logical space, as we noted above in Section 2.1, to either contextual realist approaches—and so to violations of Leibnizianity—or to approaches that forego some aspect of realism about the quantum state. The key claim of Schmid et al. is that they identify that it is indeed possible to have a Leibnizian realist interpretation of quantum theory, but only so long as the causal and inferential components are inherently nonclassical. While they do not provide any great amount of detail, they note that quantum causal modelling [52,53,54,55] is likely to play a significant role in the development of any such realist interpretation, as well as more abstract characterisations of probability theory [69]. While the sentiment of this project is laudable, I make merely two cautionary comments in passing. As a rejoinder to the involvement of quantum causal modelling, it seems as though the Shrapnel–Costa theorem, which itself is built on the principles of quantum causal modelling, will make it difficult for any such generalisation on its own to salvage noncontextuality. On the involvement of abstract characterisations of probability theory, any generalisation of probability theory that salvages noncontextuality that is non-Kolmogorovian faces the contention—brought by Feintzeig and Fletcher [70]—that it simply does not offer any clear advantage, in terms of, say, guiding rational action, over contextual theories with ordinary Kolmogorovian probability.

The second remaining option for rescuing Einstein–Bell realism is arguably the more interesting for proponents of causal symmetry. This option would entail biting the bullet on contextuality, and either (i) considering the contextuality merely apparent and finding some natural explanation for it in terms of noncontextual ontic structure and some epistemic constraint, or (ii) accepting that quantum theory is underpinned by contextual ontic structure and then accounting for how the world might conspire to render this contextuality operationally undetectable in a classical setting (or, perhaps even both (i) and (ii)). The former, of course, is the method by which causally symmetric approaches meet the challenge of the Kochen–Specker theorem and the ontological models framework. The natural explanation there is that an observer’s ordinary temporal orientation constrains epistemic access to the future causes that influence the properties of some quantum system’s beables, and so make these ultimately noncontextual hidden variables seem contextual from the observer’s perspective. The ideal response for proponents of causal symmetry in the current scenario would be to identify a corresponding epistemic, or otherwise, constraint that shows how apparent contextuality can arise from actual noncontextuality. While I do not have a definitive argument to demonstrate the plausibility of such an account, I would like to make a speculative suggestion for the sort of constraint and corresponding consequences that might be required for this task.

However, before I do, it is worth pushing the dialectic of this work towards the following inconvenient conclusion. If the primary motivation for adopting a causally symmetric framework is to rescue Einstein–Bell realism, then we have just seen that the Shrapnel–Costa theorem renders this task either impossible, or at best beholden to the possibility of some further account explaining how, say, apparent contextuality arises from some noncontextual footing. However, and importantly, even if such an account could be found, it still may not be enough to rescue Einstein–Bell realism. Whether it does or not hangs on how ‘natural’ the account is. As we saw in Section 2.2, one of the strengths of causally symmetric approaches that rescue Einstein–Bell realism from the traditional no-go theorems is that the ideology of causal symmetry is more economical than a rejection of classical ontology. However, it is difficult to see how any account that introduces potentially artificial constraints or complex mechanisms can be proposed without significantly reducing the ideological economy of causal symmetry, jeopardising the very grounds upon which one might consider the approach more virtuous [1].

Thus, in response to the Shrapnel–Costa theorem, the outlook is particularly worrying for Einstein–Bell realism and classical ontology. Not only is the obvious consequence of the theorem is a straightforward rejection of Einstein–Bell realism, but even where there looks to be a possibility of accounting for contextual ontic variables within a causally symmetric framework, the cost of such an account could result in a dramatic decrease in the ideological economy of causal symmetry, and so render the rejection of classical ontology favourable on grounds of scientific virtue. Either way, it looks very difficult to maintain a classical ontology for quantum theory.

Despite this pessimistic prognosis, I will finish this part of the dialectic with a speculative suggestion of where I see the most promising kind of possibilities for Einstein–Bell realism in the face the Shrapnel–Costa theorem. In [3] we considered some peculiar problems faced by what we call the Price–Wharton approach to causally symmetric local hidden variables in the context of a universal ψ-epistemic wavefunction (which we refer to as Ψ-epistemic quantum cosmology). The problem is the following. The Price–Wharton approach poses a two-time boundary value problem for the kinematics of the underlying hidden variables. Additionally, as a result of the traditional no-go theorems, initial data on a Cauchy surface forming a well-posed Cauchy problem and comprised of local, noncontextual classical variables cannot possibly recover the statistical predictions of quantum theory. This is despite the fact that the initial data about the quantum state on the same Cauchy surface perfectly well comprise a well-posed Cauchy problem, due to the parabolicity of the Schrödinger equation. That the ontic state variables on the initial Cauchy surface do not comprise a well-posed Cauchy problem should be expected, though, as a two-time boundary problem requires additional information from the final boundary to obtain a complete determination between the boundaries. So sufficient initial data on a Cauchy surface to form a well-posed Cauchy problem would lead to an overdetermination of the ontic state at the final boundary. One way around this that we consider is that the laws governing the ontic state variables cannot be parabolic or hyperbolic PDEs.

However, there is an alternative, more speculative possibility that we briefly consider in this context, albeit one that is set to one side in [3]. The ontic state variables do solve a Cauchy problem, but are also determined as a two-time boundary problem (so as to circumvent the no-go theorems). The tension generated by this overdetermination is addressed by a constraint on the free action of an agent controlling the nature of the data on the initial and final boundary. We put it as follows:

the tension that would need to be resolved is between: (i) the solution of a Cauchy problem from freely, arbitrarily and (ideally) completely specifiable initial data; and (ii) the symmetric expectation that the final boundary be equally freely, arbitrarily and completely specifiable. One way to escape this tension would be to remove the freedom to completely specify data on the final boundary: an agent controlling the final boundary would just happen to ‘choose’ a measurement that concords with the deterministic evolution of the ontic state. However, this would break the symmetry between the final and initial boundaries and would also remove the element of control that renders the Price–Wharton picture causally symmetric. To retain the symmetry would thus require some as-yet-unspecified principled constraint that limits an agent’s ability to freely, arbitrarily and completely specify both initial and final boundary data. This constraint must be such that the aspects of the ontic state on the *initial* Cauchy surface that are a consequence of the choices specified at the *final* boundary are not epistemically accessible before the final boundary is specified—and vice versa.

[3] (p. 7)

The significance of this possibility for the dialectic around the Shrapnel–Costa theorem is the following. Consider the nature of this proposed constraint on an agent’s ability to control the boundary data. One way for this constraint to work would be to limit agential control over the complete specifiability of data on both the initial and final boundary (in the same way as one can perform quantum measurements, but cannot control the precise outcome). Another way for this constraint to work would be to limit, say, the time intervals at which an agent can specify data on initial and final boundaries. Without such a temporal constraint, we should expect the data on the initial and final boundaries in a two-time boundary value account to be generally related by elliptic PDEs. However, one could imagine, albeit in a highly speculative fashion, that if the distance between the boundaries were constrained to certain discrete time intervals—perhaps frequent enough to be practically undetectable to the agent—the initial and final ontic state variables could be related consistently by some hyperbolic PDE. (One could think of this as roughly analogous to turning a ‘thick’ sandwich problem into a ‘thin’ sandwich problem in geometrodynamics [71,72,73]. This problem in geometrodynamics is motivated by the corresponding problem in electrodynamics, where the free specification of data on two boundaries with periodic boundary conditions can be made consistent by stipulating that the interval of time between the two boundaries is an integer multiple of a half period of the harmonic oscillation of the field [72] (p. 355)). This would apparently resemble a well-posed Cauchy problem over the ontic state variables on the initial boundary, but those variables would be at least partly determined by the ontic state on the final boundary. There would be overdetermination in such a case, but the overdetermination would be fine-tuned so as not to create a contradiction. An agent would then be similarly constrained to intervene on a system at specific times on pain of generating a contradiction in the hidden variables that describe the kinematical properties of some quantum system between the boundaries.

This speculative example is interesting because of its potential realisation of process contextuality. According to process contextuality, the parts of the physical system that are contextual could include ontic states, global properties, causal mechanisms, dynamical laws, and boundary conditions. This implies that these features of the ontic process might depend upon the context of measurement. This seems to be a feature of the above example: the ontic state variables on the boundaries, and whether they are modelled by, say, elliptical or hyperbolic dynamical laws depends upon, say, the precise time intervals across which the agent is able to intervene on the system. In addition, this account looks highly fine-tuned, which also appears to be a feature of process contextuality. Could this then be a case of process contextuality?

Moreover, the imagined (and admittedly unspecified) constraint on the agent that limits the agent’s capability “to freely, arbitrarily, and completely specify both initial and final boundary data” to specific time intervals should ultimately, with the right detail, render such a causally symmetric local hidden variable approach process noncontextual, as the constraint removes the above-mentioned contextuality (but admittedly for the price of fine-tuning). This, by my lights, appears to qualify as biting the bullet on contextuality as per the Shrapnel–Costa theorem, but also as having such process contextuality be merely apparent. This is because the apparent contextuality would simply be a feature of our continuous-time model of the causally symmetric hidden variables, which themselves are determined on a discrete-time basis due to the constraint. Provided that the imagined constraint could be given a ‘natural’ explanation (which I have certainly not attempted here), the process contextuality could be accounted for in terms of noncontextual ontic structure and some agent-centred constraint. However, there must be considerable doubt concerning whether such a constraint could provide a natural explanation, at the very least because it explicitly introduces fine-tuning into the explanation of how apparent contextuality arises from a noncontextual ontic state. So long as one thinks that a Leibnizian principle of parsimony is a good guide to scientific methodology, fine-tuning of this sort is not a virtue of a theory—and it is moreover worth noting that, due to the nonextendibility result above, the addition of noncontextual ontic structure cannot provide more information than is contained in the quantum description.

I do not mention this speculative example here to argue in its favour, by any means. My purpose here is to provide a demonstration of the sort of argumentation that *would* be required as part of what is arguably the only reasonable path forward for the Einstein–Bell realist. What is required is an account of, say, apparent process contextuality that has these sorts of features. This moreover demonstrates the interrelation between the naturalness of the constraint and the ideological economy of the model: unless the constraint makes use of uncontroversial features of time, space, agency, and so on, it will face a hard task keeping the ideology economical.

## 5. Final Thoughts

Is this the end of a classical ontology for quantum mechanics? Yes, I think this has to be the end. Perhaps there is hope that locality and noncontextuality can be salvaged by an appropriate generalisation of probability theory and the characterisation of inference. However, the path ahead for the causal symmetry program looks less hopeful. While there is an open logical possibility that a causally symmetric hidden variable approach might be able to provide a natural explanation of how a noncontextual ontic state might appear to be contextual on account of some low-cost constraint, this seems at the present moment highly unlikely. What is more, the nonextendibility result implies that any noncontextual ontic state cannot contribute to the predictive power of quantum mechanics—any such contribution can only be made by additional instrument-contextual variables. So any such logical possibility is unlikely to render accepting causal symmetry more economical than rejecting a classical ontology, and will necessarily fail to contribute to increased predictive power for quantum mechanics. In so far as this unlikely logical possibility is the last refuge for Einstein–Bell realism, it looks like we should give up on Einstein–Bell realism and, with it, classical ontology.
